# The Pseudomonas aeruginosa PilSR Two-Component System Regulates Both Twitching and Swimming Motilities

**DOI:** 10.1128/mBio.01310-18

**Published:** 2018-07-24

**Authors:** Sara L. N. Kilmury, Lori L. Burrows

**Affiliations:** a Department of Biochemistry and Biomedical Sciences, McMaster University, Hamilton, ON, Canada; b Michael G. DeGroote Institute for Infectious Disease Research, McMaster University, Hamilton, ON, Canada; University of Washington

**Keywords:** cell surface, cystic fibrosis, flagellar gene regulation, two-component regulatory systems, type IV pili

## Abstract

Motility is an important virulence trait for many bacterial pathogens, allowing them to position themselves in appropriate locations at appropriate times. The motility structures type IV pili and flagella are also involved in sensing surface contact, which modulates pathogenicity. In Pseudomonas aeruginosa, the PilS-PilR two-component system (TCS) regulates expression of the type IV pilus (T4P) major subunit PilA, while biosynthesis of the single polar flagellum is regulated by a hierarchical system that includes the FleSR TCS. Previous studies of Geobacter sulfurreducens and Dichelobacter nodosus implicated PilR in regulation of non-T4P-related genes, including some involved in flagellar biosynthesis. Here we used transcriptome sequencing (RNA-seq) analysis to identify genes in addition to *pilA* with changes in expression in the absence of *pilR*. Among the genes identified were 10 genes whose transcription increased in the *pilA* mutant but decreased in the *pilR* mutant, despite both mutants lacking T4P and pilus-related phenotypes. The products of these inversely dysregulated genes, many of which were hypothetical, may be important for virulence and surface-associated behaviors, as mutants had altered swarming motility, biofilm formation, type VI secretion system expression, and pathogenicity in a nematode model. Further, the PilSR TCS positively regulated transcription of *fleSR*, and thus many genes in the FleSR regulon. As a result, *pilSR* deletion mutants had defects in swimming motility that were independent of the loss of PilA. Together, these data suggest that in addition to controlling T4P expression, PilSR could have a broader role in the regulation of P. aeruginosa motility and surface sensing behaviors.

## IMPORTANCE

Surface appendages such as type IV pili and flagella are important for establishing surface attachment and infection in a host in response to appropriate cues. The PilSR regulatory system that controls type IV pilus expression in Pseudomonas aeruginosa has an established role in expression of the major pilin PilA. Here we provide evidence supporting a new role for PilSR in regulating flagellum-dependent swimming motility in addition to pilus-dependent twitching motility. Further, even though both *pilA* and *pilR* mutants lack PilA and pili, we identified sets of genes downregulated in the *pilR* mutant and upregulated in a *pilA* mutant as well as genes downregulated only in a *pilR* mutant, independent of pilus expression. This finding suggests that change in the inner membrane levels of PilA is only one of the cues to which PilR responds to modulate gene expression. Identification of PilR as a regulator of multiple motility pathways may make it an interesting therapeutic target for antivirulence compounds.

## INTRODUCTION

Prokaryotes rely on the use of two-component systems (TCSs) to control many of their cellular activities. Typically comprised of a membrane-bound histidine sensor kinase and a cytoplasmic response regulator, TCSs allow bacteria to respond rapidly to chemical and physical changes in their intra- or extracellular environments, altering expression of specific genes in response to a stimulus (1). The opportunistic pathogen Pseudomonas aeruginosa encodes a higher-than-average number of TCSs (2) that control diverse functions, including several motility phenotypes. Flagellum-dependent swimming motility, for example, is controlled through a regulatory cascade that includes the transcriptional regulator FleQ (3) and the FleS-FleR TCS, which like many TCSs also requires the alternate sigma factor RpoN (σ^54^) (4). FleQ controls transcription of *fleS-fleR* in addition to other flagellar, adhesion, and biofilm-associated genes, in a c-di-GMP-dependent manner (3, 5). FleSR is implicated in the expression of 20 or more flagellar biosynthetic genes in P. aeruginosa, as well as additional genes not previously known to be involved in flagellar assembly or function (6).

The other major motility system in P. aeruginosa is the type IV pilus (T4P) system, which is used for twitching across solid and semisolid surfaces (7, 8) among other important functions. In contrast to the single polar flagellum that is used to propel the cell in low-viscosity media, the cell extends multiple pili that retract either independently or in a coordinated bundle, pulling it toward the point of attachment (9–11). Pili can be extended from either pole, but typically, a single pole is used at one time, allowing for directed movement (12). The majority of the pilus fiber is made of hundreds to thousands of subunits of the major pilin protein, PilA (13), the expression of which could be energetically costly to the cell if not tightly controlled.

*pilA* transcription is regulated by another TCS, PilS-PilR, in P. aeruginosa and many other T4P-expressing bacteria (14–18). PilS is an atypical sensor histidine kinase (SK) with six transmembrane segments (19–21) that allow PilS to interact directly with PilA for pilin autoregulation (22). PilR is the cytoplasmic response regulator (RR) that binds with σ^54^ to the *pilA* promoter to activate transcription (23, 24). Neither *pilA* mutants nor *pilR* mutants express PilA or T4P, but they have opposite PilR activation states, in that *pilR* mutants have no activity, while *pilA* deletion results in PilR hyperactivity (22, 23). Activation of PilR upon transient decreases in intracellular PilA levels may be one way in which pilus attachment events are detected.

In contrast to the response regulator FleR, which has a well-defined regulon in P. aeruginosa (6), the suite of genes potentially controlled by PilR is poorly characterized. Genetic and *in silico* analyses of the PilR regulons of Geobacter sulfurreducens (16, 25) and Dichelobacter nodosus (17) have been performed, but comparable studies of P. aeruginosa are lacking. Screening of the G. sulfurreducens genome for putative PilR binding sites revealed 54 loci with predicted σ^54^-dependent promoters, many of which were upstream of genes for T4P and flagellar biosynthesis or for cell wall biogenesis (25). Those data, in combination with work performed with D. nodosus, which identified several surface-exposed proteins whose expression was controlled by PilR (17), suggest that P. aeruginosa PilR likely has additional functions beyond control of *pilA* transcription. However, each of the studies cited focused mainly on identification of genes and characterization of their pilus-related functions without examining other phenotypic consequences of loss of *pilR*.

In this work, we used transcriptome sequencing (RNA-seq) analysis to identify genes that were dysregulated by loss of *pilR*. Because *pilR* mutants lack pili, which are important for surface sensing (26) and control of downstream events such as biofilm formation (27), we included a *pilA* mutant in our analysis to distinguish genes whose expression is specifically controlled by PilR from those that are affected by the loss of PilA. In addition to several genes that were coregulated with *pilA*, which we have termed “pilin-responsive” genes, we also identified multiple flagellar genes, including the FleSR TCS, as being downregulated only in the absence of *pilR*, in a pilin-unresponsive manner. We show that the consequence of this downregulation is a previously unreported defect in swimming motility in both *pilS* and *pilR* mutants, independent of the loss of PilA. This work defines the pilin-dependent and -independent regulons of PilR and provides evidence for a direct regulatory connection between the P. aeruginosa T4P and flagellar motility systems.

## RESULTS

### The expression of multiple genes is similarly altered in *pilA* and *pilR* mutants.

We performed RNA-seq analysis to identify genes in addition to *pilA* that might be controlled by the PilSR TCS. However, in designing this experiment, we considered the following. (i) *pilR* mutants also lack expression of PilA. (ii) Loss of PilA contributes to a decrease in intracellular levels of the messenger molecule cyclic AMP (cAMP) (28). (iii) There are more than 200 genes in P. aeruginosa that are at least partially cAMP dependent, including Vfr, a cAMP-binding virulence factor regulator (28). To separate genes that are affected by the loss of PilA that occurs in both *pilA* and *pilR* mutants from the genes that are truly regulated by PilR, we categorized genes as those whose expression was changed in only the *pilA* or *pilR* backgrounds, versus both backgrounds, compared to the wild-type (WT) PAK strain (see Fig. S1 in the supplemental material). The former group may also include genes that are cAMP dependent. We did not include a *pilS* mutant in RNA-seq analysis, because PilS potentially interacts with alternate response regulators, making it more challenging to distinguish genes that are controlled by PilSR and those regulated by PilS and other unidentified RRs (29). Genes that were dysregulated similarly by at least twofold in both the *pilA* and *pilR* mutants are summarized in Table S1 and included several T4P-associated genes such as *tsaP* (30), and minor pilin genes *fimU*, *pilV*, *pilW*, *pilY1*, and *pilE* (31), previously identified as being Vfr dependent (28). In total, 18 of 56 genes in this category (shown on a gray background in Table S1) are also Vfr and cAMP dependent (28). Since the expression of genes in this class was affected by the loss of PilA, suggesting PilR’s role is indirect, they were not examined further. No genes whose expression was dysregulated by loss of *pilA* but not *pilR* were identified.

10.1128/mBio.01310-18.1 FIG S1  RNA-seq experimental design. Our RNA-seq experiment was designed to distinguish between genes dysregulated by loss of *pilR* versus loss of *pilA*, as *pilR* mutants also lack the pilin protein. Genes may be coordinately dysregulated in both *pilA* and *pilR* mutants compared to the WT due to loss of PilA or pilus expression. Genes that are inversely dysregulated in *pilA* and *pilR* mutants are upregulated in the absence of PilA (pilin responsive) in a *pilR-*dependent manner. Genes dysregulated by loss of *pilR*, but not *pilA*, are dependent only on PilR expression and referred to as pilin unresponsive in this study. Download FIG S1, TIF file, 2.8 MB. Copyright © 2018 Kilmury and Burrows. 2018 Kilmury and Burrows This content is distributed under the terms of the Creative Commons Attribution 4.0 International license.

10.1128/mBio.01310-18.4 TABLE S1  Genes similarly dysregulated in *pilA* and *pilR* mutants. Download TABLE S1, DOC file, 0.1 MB. Copyright © 2018 Kilmury and Burrows. 2018 Kilmury and Burrows This content is distributed under the terms of the Creative Commons Attribution 4.0 International license.

### Ten genes are inversely dysregulated by loss of *pilA* versus *pilR*.

The expression of a subset of 10 genes was decreased in the *pilR* mutant but markedly increased in the *pilA* mutant, even though both mutants fail to express PilA (23) (Table 1 and Table S2). We categorized these genes as “pilin responsive,” because similar to PilA, their expression was dependent on PilR and increased when PilA levels were low. All but three of these genes encode hypothetical proteins or are unannotated in the *P*. *aeruginosa* PAO1 genome. The coregulation of these genes with *pilA* suggests that their products could be previously unidentified contributors to T4P biogenesis and/or function or to other forms of motility.

10.1128/mBio.01310-18.5 TABLE S2  Genes inversely dysregulated in *pilA* and *pilR* mutants. Download TABLE S2, DOC file, 0.1 MB. Copyright © 2018 Kilmury and Burrows. 2018 Kilmury and Burrows This content is distributed under the terms of the Creative Commons Attribution 4.0 International license.

**TABLE 1:**
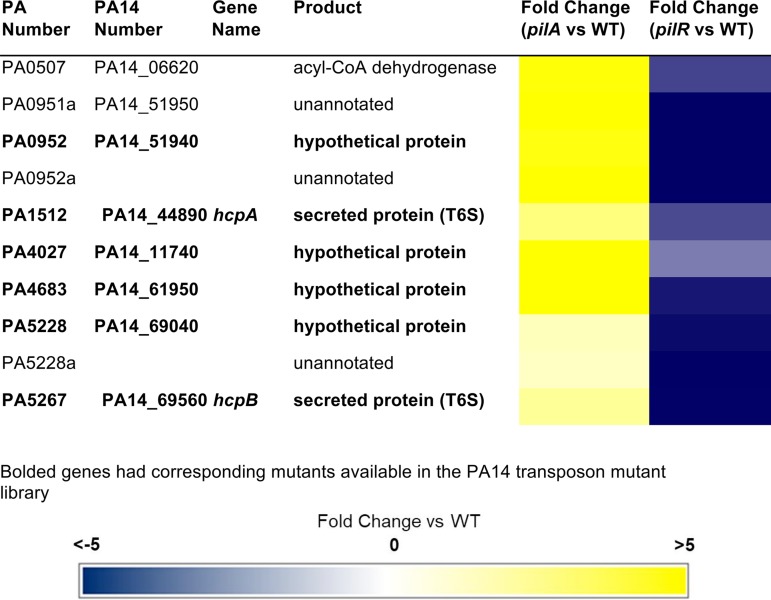
Genes inversely dysregulated by *pilA* and *pilR*^a^

a Boldface genes are associated with the flagellar system.

**FIG 1:**
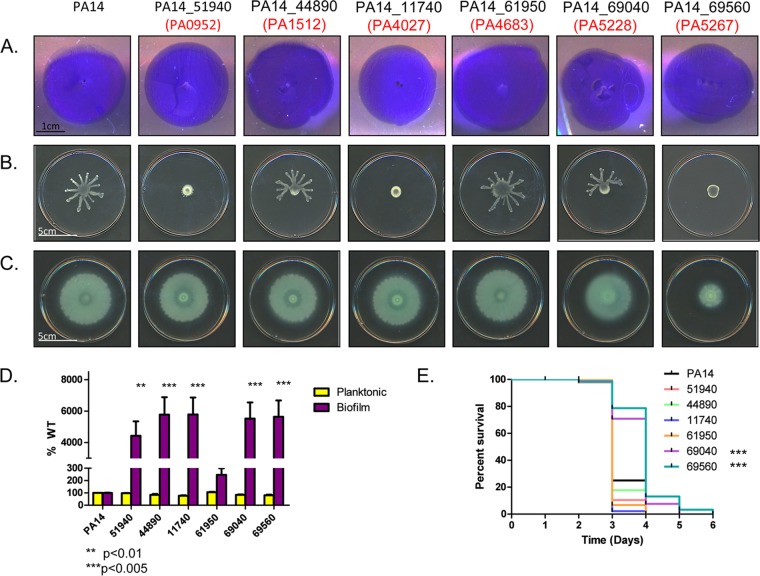
PA14 mutant homologs of inversely dysregulated genes affect motility phenotypes. (A to E) Available PA14 transposon mutant homologs of inversely dysregulated genes identified in strain PAK were tested for twitching (A), swarming (B), and swimming (C) motilities, biofilm formation (D), and pathogenicity toward *Caenorhabditis elegans* (E) to identify the functions of hypothetical proteins. All mutants displayed WT twitching, but only a subset had defects in swimming and/or swarming motility. Several mutants exhibited a hyperbiofilm phenotype, while two had defects in *C. elegans* killing (***, *P* < 0.005). For the motility assays, images representative of three independent experiments are shown.

To test this hypothesis, we extracted mutants with insertions in homologs of those PAK genes from the ordered *P*. *aeruginosa* PA14 transposon (Tn) library (32). There were no transposon insertions in three of the ten genes, and one additional mutant failed to grow in liquid culture. The PAO1 and PA14 designations for the remaining six genes of interest for which mutants were available are listed in Table 1. We tested these mutants for twitching, swimming, and swarming motilities. While all had WT twitching motility (Fig. 1A), insertions in PA14_51940 (PA0952), PA14_11740 (PA4027), and PA14_69560 (PA5267, *hcpB*) caused defects in swarming motility. PA14_44890 (PA1512) and PA14_69040 (PA5228) mutants had altered swarming motility, suggesting that coordinated motion is disrupted (Fig. 1B). Disruption of PA14_695060 (*hcpB*) also reduced swimming, alluding to a role in flagellar function or biosynthesis, in addition to its established function in type VI secretion (Fig. 1C). Together, these data indicate that genes coregulated with *pilA* are not necessarily required for T4P function. However, a subset of these genes are involved in other forms of motility, biofilm formation (Fig. 1D), and pathogenicity in *Caenorhabditis elegans* (Fig. 1E). The phenotypes of these mutants are interesting and the focus of ongoing studies.

### A subset of genes are dysregulated only by loss of PilR.

In the *pilR* mutant, 89 genes were dysregulated ≥2-fold (Table S3). To prioritize our follow-up studies, we focused on a shorter list of genes with ≥3-fold changes in expression. Of particular interest were 24 genes whose expression was ≥3-fold altered in *pilR* mutants but unaffected by the loss of *pilA*. These *pilR-*dependent but pilin-unresponsive genes are highlighted in Table 2. According to the *Pseudomonas* genome database (33), these genes include five putative chemotactic transducers, two biofilm-associated chemosensory proteins, six hypothetical proteins, and several metabolic enzymes. However, motility-associated genes were the most common class identified. The genes encoding the T4P assembly ATPase, PilB, and prepilin peptidase, PilD, which share a divergently oriented promoter with *pilA*, were downregulated in *pilR* mutant but unaffected by the loss of *pilA* (Table 2), even though previous studies suggested that they were controlled by σ^70^, not PilSR and σ^54 ^(34).

10.1128/mBio.01310-18.6 TABLE S3  Genes dysregulated by the loss of *pilR* only. Download TABLE S3, DOC file, 0.2 MB. Copyright © 2018 Kilmury and Burrows. 2018 Kilmury and Burrows This content is distributed under the terms of the Creative Commons Attribution 4.0 International license.

**TABLE 2:**
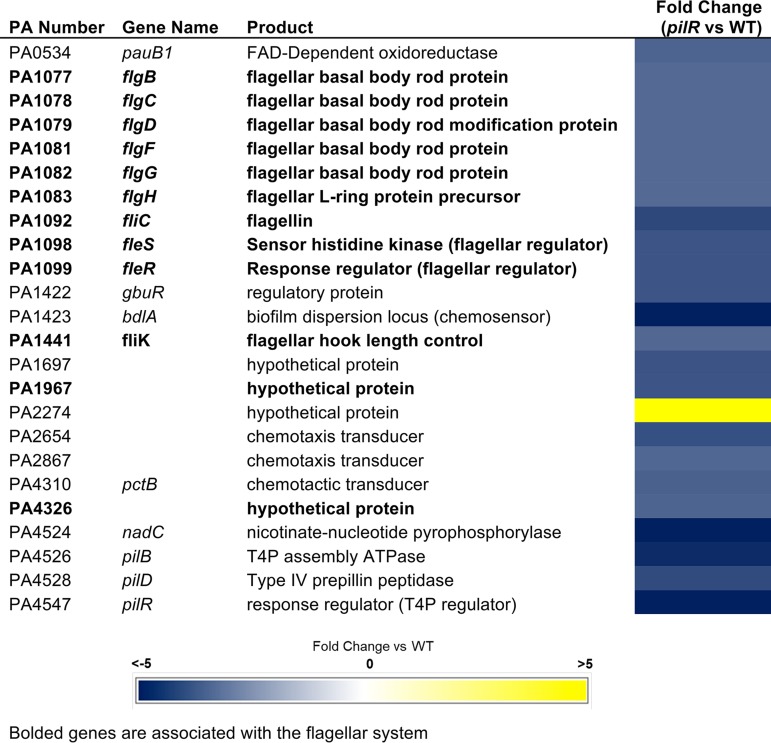
Pilin-unresponsive, *pilR*-dependent genes

### Multiple flagellum biosynthetic genes are downregulated in a *pilR* mutant.

In addition to the T4P-associated genes above, several flagellum biosynthetic genes had decreased expression only in the *pilR* background (Table 2, boldface text; Table S3). Among them were *fleS-fleR* encoding the FleSR TCS, part of a regulatory cascade that controls the expression of genes associated with flagellum biosynthesis and function, including two FleR-dependent genes that encode proteins of unknown function, PA1967 and PA4326 (4, 6). Each had approximately threefold-lower expression in the *pilR* mutant compared to the WT, while there was no difference in their expression in *pilA* mutant versus WT. This trend was verified using reverse transcription-PCR (RT-PCR), though the magnitude was closer to twofold by this method (Fig. S2). Of the flagellar genes in this category (Table 2), 10 of 12 (excluding *fleS* and *fleR*) are *fleR* dependent (6). The remaining two, *fliE* and *fliF*, are known to be FleQ dependent, but have also been shown to have decreased (≥2-fold) transcription in a *fleR* mutant in both this (Table S3) and a previous study (6). These data suggest that PilSR positively regulates *fleSR* expression and that when PilR is absent, expression of FleSR-dependent genes is decreased accordingly.

10.1128/mBio.01310-18.2 FIG S2  RT-PCR validation of *fleSR* transcription levels. *fleS-fleR* were downregulated approximately twofold in *pilS* and *pilR* mutants but not in *pilA* mutant. Means and standard errors from three independent experiments are shown. Download FIG S2, TIF file, 2.6 MB. Copyright © 2018 Kilmury and Burrows. 2018 Kilmury and Burrows This content is distributed under the terms of the Creative Commons Attribution 4.0 International license.

### Swimming motility is impaired by the loss of *pilS*-*pilR*.

**FIG 2:**
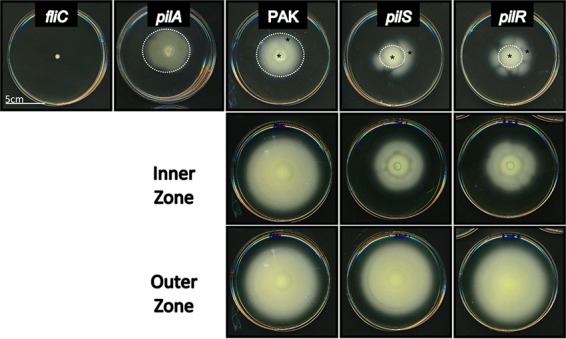
Swimming motility is impaired in *pilS* and *pilR* mutants. Loss of *pilS* or *pilR* results in decreased swimming motility (53.7% ± 0.5% and 47% ± 1.4% of WT, respectively; *P* < 0.005) in a plate-based assay. *pilA* mutants swim comparably to the WT, indicating that the swimming defect is not PilA dependent. *pilS* and *pilR* mutants appear to acquire suppressors that overcome these defects resulting in asymmetrical flares. Reinoculation of swimming plates with cells from the interior of swimming zones—inside the white dotted circles of *pilSR* (inner) recapitulate the original phenotype, while cells taken from the flares (outer; from flares outside the white dotted circles, except for WT) swim to WT levels. Black asterisks denote the locations where cells were taken for the reinoculated swimming plates.

Because PilSR regulates *fleSR* and their regulon, we tested whether *pilS* and *pilR* had defects in swimming motility. A *fliC* mutant lacking the flagellin subunit was used as a negative control. *pilA* mutants swam comparably to WT PAK, while *pilS* and *pilR* mutants—which also lack surface pili—exhibited significant swimming defects (*P* < 0.005), with uniform zones that reached about 53.7% ± 0.5% and 47% ± 1.4% of WT, respectively (standard error, *n* = 3) (Fig. 2, white dotted lines). Interestingly, both *pilS* and *pilR* mutants produced flares with increased motility extending beyond these uniform swimming zones. These flares were hypothesized to be the result of suppressor mutations that could overcome the effect of *pilS* or *pilR* deletion on swimming.

To test this idea, we isolated cells from the inner swimming zones of *pilS* and *pilR* plates (inside the white dotted lines in Fig. 2) and the putative suppressor mutants (flares outside the white dotted lines) and reassessed their ability to swim after culturing them overnight. For controls, we took samples from the WT zone close to the point of inoculation (“inner”) and from the outer edge of the swimming zone (“outer”). Repeating the swimming assays with these samples revealed no difference in swimming between inner and outer samples from WT. However, *pilS* and *pilR* cells taken from the inner swimming zones recapitulated the original swimming motility defects of the mutants and similarly produced highly motile suppressors. Meanwhile, cells taken from the outer flares had motility comparable to that of the WT (Fig. 2), indicating that they likely retained a suppressor mutation(s) that allows for full motility in the absence of *pilSR*.

To test whether other flagellum-dependent phenotypes were affected by the loss of *pilSR*, we measured swarming motility, using the original mutants and the suppressors isolated from the swimming experiments above. *pilSR* mutants of strain PAO1 were previously reported to be nonswarmers (35), but in our hands, the same mutants in the PAK background retain partial swarming motility, albeit with an altered morphology compared to WT. The PAK *pilSR* mutants swarmed similarly to a *pilA* mutant, with fewer and irregular tendrils (Fig. S3). Interestingly, *pilSR* mutants isolated from the outer flares of the swimming plates in Fig. 2 had swarming motility comparable to those isolated from the inner zones and the *pilS* and *pilR* parent strains. While flagella are required for swarming, the suppressor mutations that restored swimming motility in the *pilS* and *pilR* backgrounds did not restore swarming, suggesting that expression of distinct swarming-related genes remains dysregulated. Investigation of the contributions of *pilSR* suppressors to rhamnolipid production, flagellar localization, and other swarming-related phenotypes will be the focus of future work.

10.1128/mBio.01310-18.3 FIG S3  Putative suppressors that restore swimming do not affect swarming motility in *pilSR* mutants. Cells from *pilS* and *pilR* mutants with putative suppressor mutations that restore swimming motility (outer) were inoculated in a swarming assay for comparison to *pilS* and *pilR* mutants that do not have suppressors (inner). Strains with possible suppressors still exhibit *pilS* and *pilR-*like swarming motility patterns, indicating that the *pilSR* phenotype is dominant. Download FIG S3, TIF file, 5.5 MB. Copyright © 2018 Kilmury and Burrows. 2018 Kilmury and Burrows This content is distributed under the terms of the Creative Commons Attribution 4.0 International license.

### FleSR impact twitching motility and *pilA* expression.

**FIG 3:**
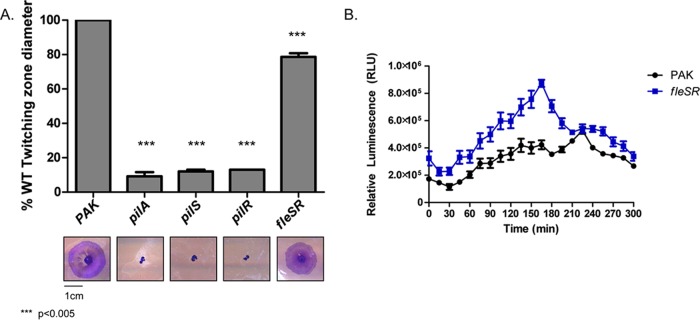
Loss of *fleSR* reduces twitching motility but increases *pilA* transcription. (A) Loss of *fleSR* reduces twitching motility by approximately 20%. Means plus standard errors (error bars) for six independent replicates are shown. Statistical significance was determined by one-way ANOVA (***, *P* < 0.005). (B) A *lux-pilA* luminescent reporter assay measuring *pilA* promoter activity indicated that *pilA* transcription is increased over 5 h. Means ± standard errors for four biological replicates are shown. RLU, relative luminescence units.

RNA-seq analyses revealed that PilR was required for WT expression of *fleSR*. We next tested whether this was a reciprocal regulatory pathway in which FleSR might contribute to regulation of *pilS-pilR* and the PilSR regulon. We tested whether loss of *fleSR* affected *pilA* expression and/or T4P function. A double deletion of *fleSR* was made in the PAK background, and twitching motility was measured. Loss of *fleSR* reduced twitching motility to a modest but significant extent (*P* < 0.005), with the double mutant reproducibly twitching to approximately 80% of WT (Fig. 3A). Interestingly, when *pilA* transcription was monitored using a *lux-pilA* reporter assay, *fleS-fleR* mutants had increased *pilA* transcription compared to the WT over a 5-h time course (Fig. 3B). Therefore, while FleS-FleR are involved in the modulation of twitching motility and *pilA* transcription, it is not yet clear whether this occurs directly through regulation of *pilSR*, as increased levels of PilA can inhibit PilSR activation (22).

## DISCUSSION

Two-component systems control a multitude of phenotypes, allowing for quick responses to sudden changes in a bacterium’s intra- and extracellular environments. These systems can be important not only for survival but also for coordinating virulence programs. Most TCSs explored thus far control the transcription of multiple genes, but prior to this work P. aeruginosa PilR had only a single known target, *pilA* (23). Microarray and bioinformatic analyses of the G. sulfurreducens PilR regulon provided evidence that PilR regulates multiple genes, including those required for soluble Fe(III) uptake (a pilin-independent phenotype), flagellar assembly and function, and cell envelope biogenesis, though these predictions were not confirmed with phenotypic assays (16, 25). Here, we showed that PilR controls the expression of multiple genes, in pilin-responsive or -unresponsive modes. Dysregulating expression of select members of the P. aeruginosa PilR regulon resulted in changes in swimming, swarming, and/or twitching motility, all phenotypes associated with virulence in specific hosts (27, 36–38).

The G. sulfurreducens and D. nodosus studies cited above failed to account for the confounding variable that PilA is not expressed when *pilR* is deleted. This was an important consideration in designing our RNA-seq experiment (see Fig. S1 in the supplemental material), as loss of PilA results in decreased cAMP levels and by extension, downregulation of cAMP-dependent genes in the Vfr regulon, which includes a number of T4P-associated genes (28). This design also enabled us to further classify genes in the PilR regulon based on their responsiveness to pilin levels. As predicted, many of the genes that were similarly dysregulated by loss of both *pilA* and *pilR* are Vfr dependent (28) (Table S1). Thus, we focused instead on those genes that were dysregulated in a PilR-dependent manner and further categorized them as pilin responsive or unresponsive.

We identified 10 pilin-responsive genes with increased transcription in a *pilA* mutant but significantly decreased transcription in the absence of *pilR* (Table 2). While this expression pattern initially seemed counterintuitive, we propose that these gene products are regulated by PilS phosphorylation or dephosphorylation of PilR in response to fluctuating PilA levels. When PilA is absent, PilS phosphorylates PilR and *pilA* promoter activity is significantly increased, presumably in an attempt to replenish intracellular PilA pools (39), and this PilR activation simultaneously increases expression of other pilin-responsive genes (Table 2 and Table S2). This signaling pathway may be one way in which adherence of a pilus to a surface is detected, through transient depletion of pilin pools in the inner membrane when attached pilus filaments fail to retract.

Many genes in this pilin-responsive category encoded hypothetical proteins or were unannotated in the *P*. *aeruginosa* PAO1 and PA14 genomes; the unannotated genes may encode regulatory RNAs. We used available mutants from the PA14 Tn library to determine whether the pilin-responsive genes were required for normal T4P function. While all mutants tested had WT twitching motility, some had decreased swarming, and one (PA14_69560) had decreased swimming motility. The only genes in this group that were characterized previously are *hcpA* and *hcpB*, paralogs which encode proteins associated with the type VI secretion system (33). This finding may represent a new link between T4P, flagellar function, and type VI secretion, as the *hcpB* mutant had defects in both swimming and swarming. This connection further explains the swimming defects of *pilS* and *pilR* mutants (Fig. 2).

We also identified genes that were affected only by the loss of *pilR*, independently of PilA levels. These genes might be modulated in response to cues detected by a different, pilin-insensitive sensor kinase that can activate PilR. While the swimming and swarming motility phenotypes of *pilS* and *pilR* mutants are comparable, implying contribution of PilS to these phenotypes, further work will be required to definitively determine the role of PilS or other putative SKs in this context. Alternatively, the pilin-unresponsive genes identified here may already be expressed in the WT at levels such that further activation upon loss of *pilA* did not meet our twofold cutoff. A third possibility is that they are indirectly upregulated as a result of PilR activity on adjacent promoters. For example, among these genes were the genes encoding the T4P assembly ATPase PilB and the prepilin peptidase, PilD, which are contiguous with *pilC* encoding the platform protein; however, there were insufficient reads in our RNA-seq analysis to accurately determine *pilC* expression levels (Table 2). On the basis of this and previous studies, *pilBCD* are not cotranscribed (33, 34). *pilB* was reported to be σ^70^ dependent (34), but our data suggest that PilR remodeling of the *pilA* promoter region for transcription by the σ^54^ holoenzyme also facilitates transcription from the divergent *pilB* promoter.

Of the pilin-unresponsive genes identified, the most abundant class were involved in biosynthesis, function, and regulation of the flagellum, including *fleSR*, which appear to be regulated by both PilS and PilR (Table 2, Fig. S2, and Table S3). Most of the other genes of this class are members of the FleSR regulon (6), which suggests that they are indirectly regulated by PilR via its control of *fleSR* levels. Future work will be directed at clarifying the exact mechanism of *pilR-*related regulation of *fleSR* expression. However, the presence upstream of *fleSR* of a NifA-like 5′-TGTN_11_AGA-3′ sequence and nearby GTCT elements, hallmarks of PilR binding sites (24), suggest that PilR may directly regulate *fleSR* transcription. Swimming motility of *pilS* and *pilR* mutants was ~45 to 50% of WT, supporting our transcriptomic data (Fig. 2). By carefully analyzing the swimming data, we hypothesized that suppressor mutations could overcome the defects imposed by *pilS* or *pilR* deletion, allowing the mutants to swim normally. Preliminary sequence analyses of these suppressors showed no mutations in *fleSR*, but it may be that mutations in *fleQ*, the promoter regions of *fleSR*, or as-yet unidentified genes could increase activity or expression of *fleSR*. The as-yet unidentified suppressors appear specific for swimming motility, as swarming motility (40) of the *pilS* and *pilR* mutants and the highly motile suppressors, all of which lack PilA, was comparable to that of a *pilA* mutant (Fig. S3).

Although *pilSR* were not considered members of the FleSR regulon (6), twitching motility was modestly but reproducibly reduced to ~80% of WT in the absence of *fleSR* (Fig. 3A), while *pilA* promoter activity was increased compared to WT (Fig. 3B). This phenotype is reminiscent of mutations that inhibit pilus retraction, impairing twitching but increasing *pilA* transcription due to depletion of PilA subunits from inner membrane pools (11, 39). During prior characterization of the FleSR regulon, two new genes (PA3713 and PA1096 or *fleP*) with motility phenotypes were identified. Strains with mutations in these genes were significantly impaired in swimming, and in the case of *fleP*, twitching motility (6). FleP was proposed to control pilus length, as when it was deleted, surface pili were significantly longer than those of WT, resulting in a form of hyperpiliation (6). Decreased *fleP* expression in our *fleSR* mutants could impair twitching motility and alter *pilA* expression.

**FIG 4:**
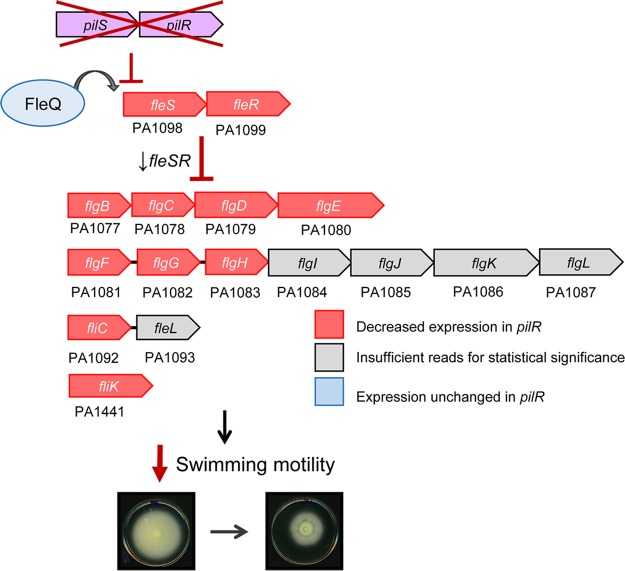
Model for *pilSR*-dependent regulation of *fleSR* and the *fleSR* regulon. Under conditions in which *pilSR* expression is decreased (low cAMP) or when PilSR is low (high intracellular PilA), *fleSR* transcription is decreased as is expression of the the *fleSR* regulon. Genes on a red background are those that had decreased expression in a *pilR* mutant in RNA-seq. Genes on a gray background had insufficient reads assigned to them from RNA-seq to accurately report differential expression. FleQ (blue) was not differentially expressed between WT or *pilR*, indicating that *pilSR* fits into the flagellar regulatory hierarchy after FleQ but before *fleSR*, as *fleQ* itself and most FleQ-dependent genes were unaffected by loss of *pilR*.

Both the PilSR and FleSR TCSs are required for full virulence of P. aeruginosa (reviewed in reference 41), as each is involved in multiple virulence-associated phenotypes. Given the overlap in phenotypes controlled by these TCSs, it is perhaps not surprising that their expression is linked. Thus, we propose a model in which PilSR positively regulates *fleSR* transcription, independently of PilA depletion (Fig. 4). Transcription of *fleSR* is predominantly dependent on FleQ (6), but since *fleQ* was not differentially expressed in *pilR*, we infer that PilSR promotes *fleSR* transcription directly, rather than by modulating FleQ expression.

Why, and under what conditions, might this regulatory circuit be active? Twitching motility is normally deployed on solid or semisolid surfaces (8), while flagella are most effective in liquid and low-viscosity conditions. One might predict that the systems are differentially activated in response to relevant environmental conditions. Instead, the regulatory integration of these two systems may be an adaptation to life as an opportunistic pathogen. T4P and flagella are typically expressed during the acute phase of infection (4, 42), and during the transition to the chronic infection phase, motility systems are downregulated in favor of those promoting type VI secretion and biofilm formation (41, 43). Clinical isolates of P. aeruginosa from chronically colonized patients are often nonflagellated and nonpiliated (44). Lack of the immunogenic flagellum may help P. aeruginosa escape phagocytosis (44), and aflagellate bacteria are better able to evade the inflammatory response of the host (45). Placing *fleSR* under control of PilSR may facilitate a more rapid transition to the chronic disease state and more efficient evasion of the host immune system. Similarly, both T4P and flagella are required for surface sensing and surface-associated behaviors such as swarming motility and activation of virulence cascades (26, 40, 46, 47). Coregulation of their expression may allow P. aeruginosa and other motile bacteria to amplify their responses to surface detection.

We identified 34 genes in addition to *pilA* whose expression was altered ≥3-fold by loss of *pilR*, 24 of which were dysregulated in a pilin-unresponsive manner, supporting previous work in G. sulfurreducens that identified putative PilR binding sites upstream of multiple genes (25). Importantly, while *pilA* and *pilR* mutants look similar with respect to their T4P-related phenotypes, their transcription profiles and other phenotypic outputs are different. For example, expression of genes encoding proteins involved in flagellum biosynthesis, including *fleSR*, are downregulated in the absence of *pilR* but unaffected by the loss of *pilA*. This work reveals a previously unappreciated regulatory connection between two diverse motility systems, with implications in detection of surface attachment and the transition from acute to chronic disease states in a host.

## MATERIALS AND METHODS

### Bacterial strains and growth conditions.

Unless otherwise specified, Pseudomonas aeruginosa PAK strains were grown in Lennox broth (LB) (Bioshop) or on LB 1.5% agar plates at 37°C. When the antibiotic kanamycin was used, it was introduced at a final concentration of 150 µg/ml. Mutants were generated by homologous recombination, using standard mating techniques described in reference 48. The strains and plasmids used in this study are shown in Table 3. Plasmids were prepared using standard cloning techniques and introduced into P. aeruginosa using electroporation.

**TABLE 3:** Strains and plasmids used in this study

Strain or plasmid	Description	Source or reference
Strains		
PAK (WT)	WT group II strain of P. aeruginosa	J. Boyd
*pilA* mutant	PAK with chromosomal deletion of *pilA*	This study
*pilS* mutant	PAK with chromosomal deletion of *pilS*	This study
*pilR* mutant	PAK with chromosomal deletion of *pilR*	This study
*fliC* mutant	PAK with FRT insertion in *fliC*	This study
*fleSR* mutant	PAK with a deletion of the full chromosomal *fleS-fleR* operon	This study
PA14 (WT)	WT group III strain of P. aeruginosa	32
PA14_51950	PA14 with a transposon insertion in gene PA14_51950	32
PA14_44890	PA14 with a transposon insertion in PA14_44890 (*hcpA*)	32
PA14_11740	PA14 with a transposon insertion in PA14_11740	32
PA14_61950	PA14 with a transposon insertion in PA14_61950	32
PA14_69040	PA14 with a transposon insertion in PA14_69040	32
PA14_69560	PA14 with a transposon insertion in PA14_69560 (*hcpB*)	32
Plasmid pMS402-p*pilA*	*pilA* promoter cloned into the BamHI site of pMS402, putting *lux* genes under control of the *pilA* promoter	22

### RNA isolation, library preparation, cDNA sequencing, and analysis.

To isolate RNA, cells from strains of interest were streaked in triplicate onto half of an LB 1.5% agar plate (100- by 15-mm petri dishes) and grown overnight at 37°C. Cells were scraped from the plates and resuspended in 1.5 ml RNAprotect bacteria reagent (Qiagen) to maintain the integrity of isolated RNA. Cells were lysed using 1 mg/ml lysozyme in 10 mM Tris-HCl and 1 mM EDTA (pH 8.0), and RNA was isolated using the RNeasy minikit (Qiagen) according to the manufacturer’s instructions. An on-column DNase treatment was performed to minimize potential DNA contamination. Purified RNA was eluted into 50 µl nuclease-free water and quantified. Quantitative reverse transcription-PCR (qRT-PCR) was performed using *rpsL* as a housekeeping gene to standardize samples by RNA content.

The following steps were performed at the Farncombe Metagenomics Facility (McMaster University, Hamilton, ON, Canada). For transcriptome sequencing (RNA-seq) analysis, rRNA was depleted from nine RNA samples (three samples from WT PAK, three samples from *pilA* mutant, and three samples from *pilR* mutant) using the Ribo-zero rRNA depletion kit (Illumina), and cDNA libraries were prepared by the NEBnext Ultra Directional library kit. Libraries were sequenced using paired-end 75-bp reads on the Illumina MiSeq platform. Reads were aligned to the PAO1 reference genome with 98% of reads mapped, and normalization and differential gene expression were calculated using the Rockhopper software (49). *q* values for each identified gene are reported in Tables S1 to S3 in the supplemental material. The complete RNA-seq data set has been deposited in NCBI GEO (accession number GSE112597).

### Twitching motility assays.

Twitching motility assays were performed as described in reference 50. Briefly, strains of interest were stab inoculated to the bottom of an LB 1% agar plate with a P10 pipette tip, and plates were incubated upside down at 37°C for 16 to 24 h. Following incubation, agar was carefully removed, and the plastic petri dish was stained with 1% crystal violet for 20 min. Excess dye was washed away with water, and twitching zone diameters were quantified using ImageJ (NIH, Bethesda, MD) (51). A one-way analysis of variance (ANOVA) statistical test was used to determine significant differences in twitching compared to WT.

### Swarming motility assays.

Swarming motility assays were performed as described in reference 52. Briefly, strains of interest were grown overnight in 5-ml LB cultures at 37°C. On the day of the assay, 0.5% agar plates with M8 buffer, supplemented with 2 mM MgSO_4_, 0.2% glucose, 0.05% l-glutamic acid, and trace metals, were prepared and allowed to solidify at room temperature for 1.5 h. Then, 3.5-µl aliquots of culture were spotted onto the center of a single plate, and the plates were incubated upright in a humidity-controlled 30°C incubator for 48 h. The plates were imaged using a standard computer scanner. Figures shown are representative of three independent experiments.

### Swimming motility assays.

Swimming motility plate assays were performed similarly to the method in reference 53, with some modifications. Overnight 5-ml cultures of strains of interest were grown at 37°C in LB with shaking. On the day of inoculation, LB 0.25% agar plates were prepared and allowed to solidify at room temperature for 1.5 h. Cell cultures were standardized to an optical density at 600 nm (OD_600_) of 1.0, and 2-µl samples were spotted onto the center of each plate. The plates were incubated upright for 16 h at 37°C, and swimming zone diameters were quantified using ImageJ (51). Where applicable, swimming zone diameters were defined at the outermost part of the swimming zone that was still uniform in appearance. Images are representative of four independent experiments. To determine statistical significance, a one-way ANOVA analysis with Dunnett’s posttest was performed, using the WT as the control strain.

### Biofilm assays.

Biofilm assays were performed similarly to the method described in reference 54, with some modifications. Briefly, P. aeruginosa strains of interest were grown in 5-ml liquid cultures of 50% LB–50% phosphate-buffered saline (PBS) (50/50 medium) overnight at 37°C with shaking. The following day, the strains were subcultured 1:25 into fresh 50/50 medium and grown to a standardized OD_600_ of 0.1. Standardized cultures were then diluted 1:500, and 150 µl of each strain of interest was plated in triplicate in a clear, 96-well plate (Nunc). The plate was closed with a 96-peg lid, providing a surface on which biofilms can form, sealed with Parafilm, and incubated with shaking for 18 h at 37°C. To quantify planktonic growth, peg lids were removed, and the 96-well plate was scanned at a wavelength of 600 nm. To quantify biofilms, peg lids were washed in PBS and stained with 0.1% crystal violet for 15 min. Following five 10-min washes in water, crystal violet was solubilized in 33% acetic acid in a fresh 96-well plate, which was scanned at 595 nm. Biofilm data were graphed as a percentage of the WT value, showing means and standard errors from three independent experiments.

### *Caenorhabditis elegans* slow killing pathogenicity assays.

Slow killing (SK) assays were performed as described previously (55). C. elegans strain N2 populations were propagated and maintained on nematode growth medium (NGM) plates inoculated with Escherichia coli Op50. The eggs were harvested to obtain a synchronized population by washing worms and eggs from NGM plates with M9 buffer. The worms were degraded by adding buffered bleach, leaving only the eggs intact. The eggs were washed with M9 buffer and resuspended in M9 buffer with rocking overnight to allow eggs to hatch into larval stage 1 (L1) larvae. Synchronized L1 worms were plated on NGM plates for 45 h to develop into L4 worms. During this process, slow killing plates supplemented with 100 µM 5-fluoro-2′-deoxyuridine (FUDR) were prepared and inoculated with 100 µl of a 5-ml LB overnight culture of bacterial strains of interest and incubated at 37°C for 16 to 18 h. Harvested and washed L4 worms (~30 to 40) were dropped by Pasteur pipette onto each SK plate. Using a dissecting microscope, plates were scored daily for dead worms, which were picked and removed. Survival curves were prepared using Graphpad Prism 5.01 (La Jolla, CA), and statistically significant differences in pathogenicity between strains were identified using Gehan-Breslow-Wilcoxon analysis.

### *pilA*-*lux* reporter assay.

Luminescent reporter assays were performed as described previously (22). Strains of interest were transformed by electroporation with the pMS402-p*pilA* plasmid, which contains the luciferase genes under control of the *pilA* promoter. Strains were grown overnight in 5-ml LB cultures supplemented with 150 µg/ml kanamycin. The following day, a 1-ml aliquot of a 1:20 dilution of cultures was prepared, and 100-µl samples were plated in triplicate in a white-walled, clear-bottom 96-well plate (Costar 3632; Corning Inc.). Luminescence and OD_600_ were measured at 15-min intervals over 5 h using a Synergy 4 microtiter plate reader (BioTek) programmed to shake continuously and incubate the plate at 37°C. Luminescence was normalized to OD_600_, and relative luminescence was plotted against time. Means and standard errors for more than four biological replicates are shown.
