# Supplementary figures and images for: The Pseudomonas aeruginosa PilSR Two-Component System Regulates Both Twitching and Swimming Motilities
Source: mBio. 2018 Jul 24;9(4):e01310-18. doi: 10.1128/mBio.01310-18 (PMC6058289; doi:10.1128/mBio.01310-18)

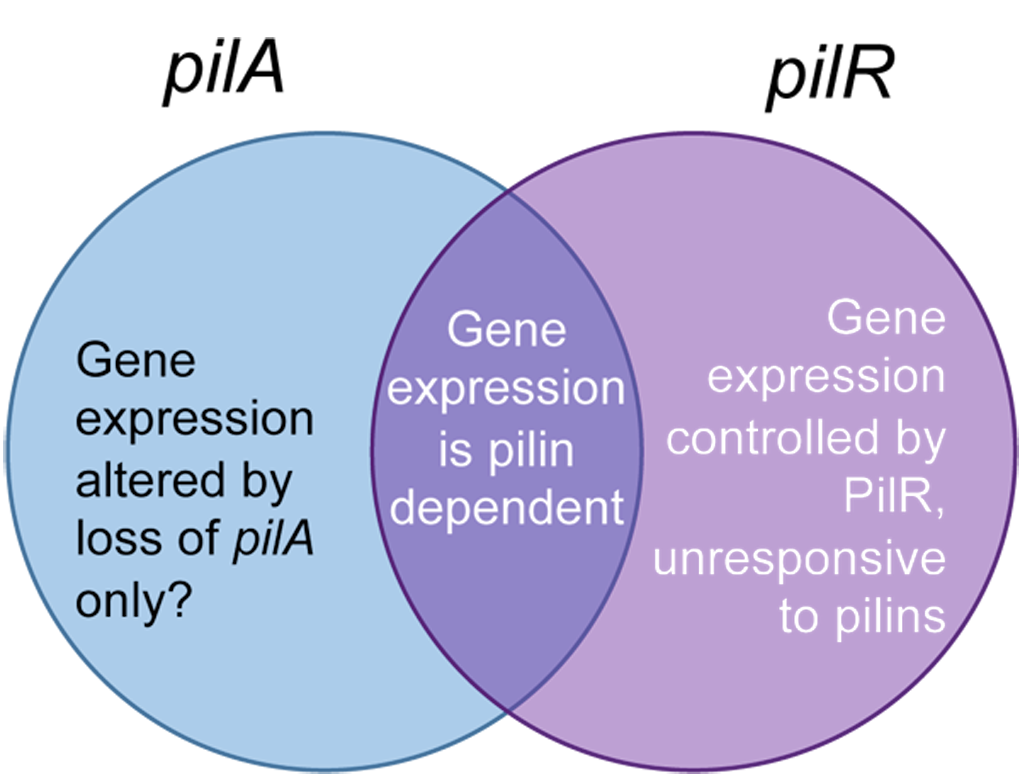

Supplement: FIG S1 [file mbo004183994sf1.tif]

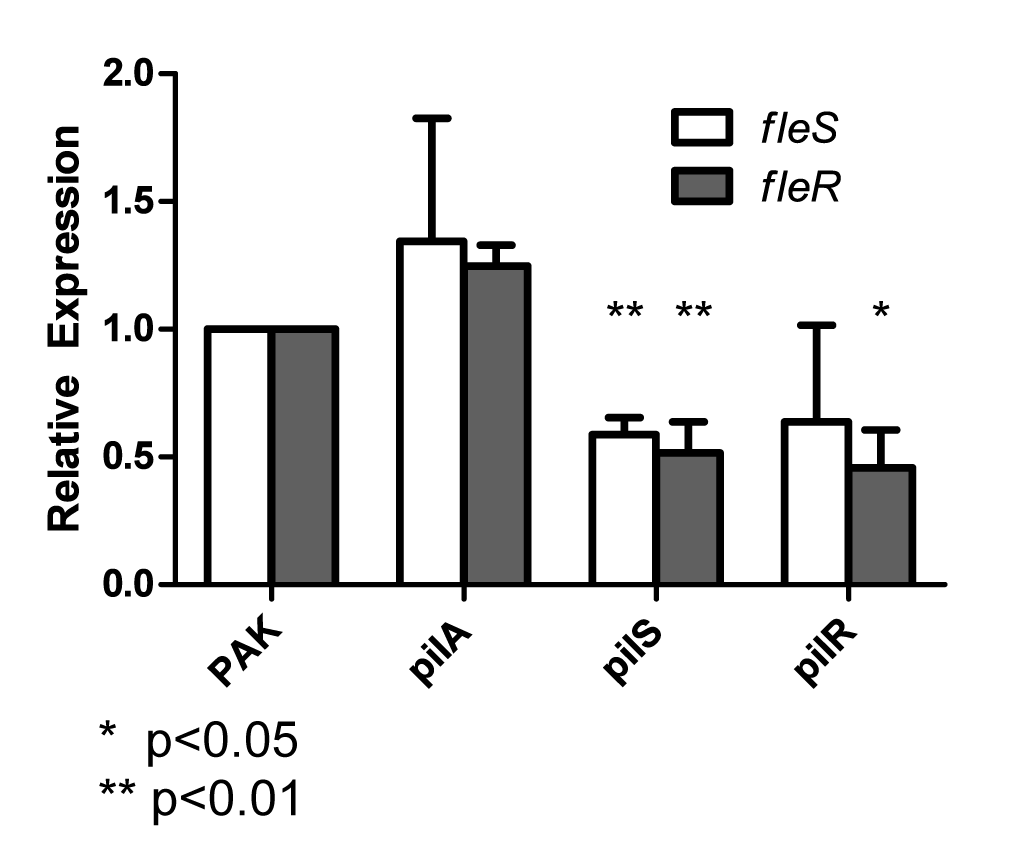

Supplement: FIG S2 [file mbo004183994sf2.tif]

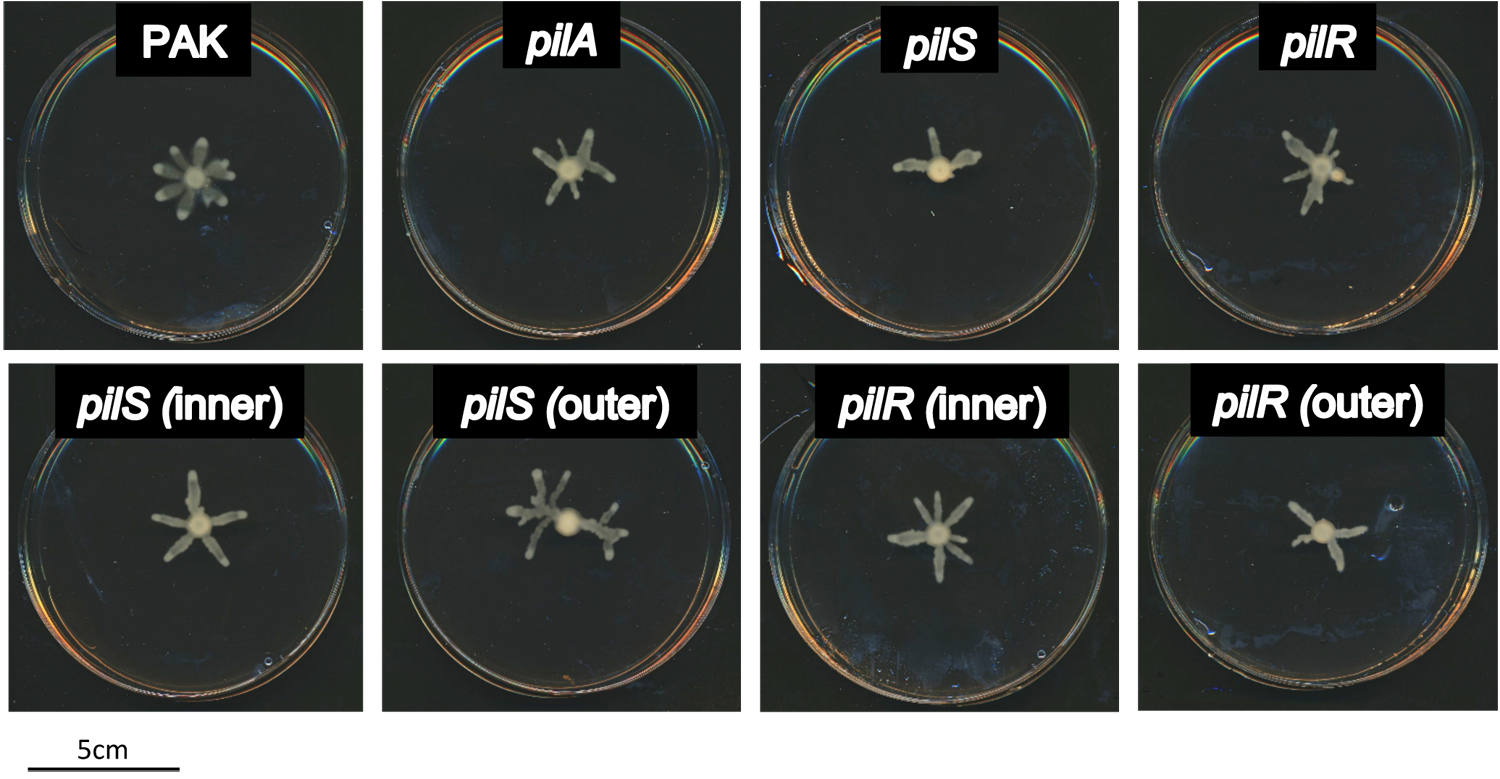

Supplement: FIG S3 [file mbo004183994sf3.tif]
