# Supplementary material for: The Pseudomonas aeruginosa PilSR Two-Component System Regulates Both Twitching and Swimming Motilities
Source: mBio. 2018 Jul 24;9(4):e01310-18. doi: 10.1128/mBio.01310-18 (PMC6058289; doi:10.1128/mBio.01310-18)
Supplement: TABLE S1 [file mbo004183994st1.doc]

*Table S1. Genes similarly dysregulated in pilA and pilR*

| **PA Number** | **Gene Name** | **Product** | **Fold Change (*pilA/*WT)** | | **Fold Change (*pilR*/WT)** | **q Value *pilA/*WT** | **q Value *pilR*/WT** |
| --- | --- | --- | --- | --- | --- | --- | --- |
| PA0020 | *tsaP* | T4P secretin-associated protein | | -2.56 | -2.65 | 0.0800 | 0.0027 |
| PA0283 | *sbp* | sulfate-binding protein precursor | | -2.32 | -3.02 | 0.0021 | 0.0000 |
| PA0284 |  | hypothetical protein | | -2.32 | -3.02 | 0.0021 | 0.0000 |
| PA0320 | *carO* | calcium-regulated OB-fold protein | | -2.40 | -3.84 | 0.0005 | 0.0000 |
| PA0391 |  | hypothetical protein | | -3.14 | -2.99 | 0.0001 | 0.0000 |
| PA0407 | *gshB* | glutathione synthetase | | -2.79 | -2.76 | 0.0015 | 0.0000 |
| PA0413 | *chpA* | component of chemotactic signal transduction system | | -2.51 | -2.39 | 0.0005 | 0.0000 |
| PA0414 | *chpB* | probable methylesterase | | -2.14 | -1.94 | 0.0133 | 0.0130 |
| PA0572 |  | hypothetical protein | | -3.76 | -3.31 | 0.0000 | 0.0000 |
| PA0602 |  | probable binding protein component of ABC transporter | | -2.73 | -3.04 | 0.0036 | 0.0000 |
| PA0613 |  | hypothetical protein | | -2.30 | -3.38 | 0.0024 | 0.0000 |
| PA0660 |  | hypothetical protein | | -2.30 | -2.01 | 0.0011 | 0.0011 |
| PA0662 | *argC* | N-acetyl-gamma-glutamyl-phosphate reductase | | -2.26 | -2.68 | 0.0015 | 0.0000 |
| PA0663 |  | hypothetical protein | | -2.02 | -2.51 | 0.0095 | 0.0000 |
| PA0664 |  | hypothetical protein | | -2.25 | -3.02 | 0.0019 | 0.0000 |
| PA0811 |  | major facilitator superfamily (MFS) transporter | | -2.33 | -2.25 | 0.0015 | 0.0001 |
| PA0812 |  | hypothetical protein | | -2.29 | -2.77 | 0.0033 | 0.0000 |
| PA0813 |  | hypothetical protein | | -2.06 | -1.90 | 0.0088 | 0.0027 |
| PA0852 | *cbpD* | chitin-binding protein | | -5.07 | -3.66 | 0.0000 | 0.0000 |
| PA0916 | *yliG* | conserved hypothetical protein | | -2.07 | -2.33 | 0.0142 | 0.0000 |
| PA0919 |  | hypothetical protein | | -2.10 | -2.22 | 0.0059 | 0.0001 |
| PA0927 | *ldhA* | D-lactate dehydrogenase | | -2.36 | -2.34 | 0.0007 | 0.0001 |
| PA1027 | *amaB* | delta1-Piperideine-6-carboxylate dehydrogenase | | -2.60 | -3.12 | 0.1270 | 0.0002 |
| PA1028 | *amaA* | L-Pipecolate oxidase | | -2.60 | -3.12 | 0.1270 | 0.0002 |
| PA1871 | *lasA* | protease | | -2.74 | -2.11 | 0.0033 | 0.0161 |
| PA2204 |  | probable binding protein component of ABC transporter | | -2.20 | -2.83 | 0.0028 | 0.0000 |
| PA2435 |  | probable cation-transporting P-type ATPase | | -2.20 | -2.21 | 0.0134 | 0.0019 |
| PA2436 |  | hypothetical protein | | -2.20 | -2.21 | 0.0134 | 0.0019 |
| PA2453 |  | hypothetical protein | | -2.14 | -3.15 | 0.0039 | 0.0000 |
| PA2782 | *bamI* | biofilm-associated metzincin Inhibitor | | -11.07 | -5.70 | 0.0000 | 0.0000 |
| PA2783 | *mep72* | putative secretion protein | | -12.17 | -12.17 | 0.0000 | 0.0000 |
| PA2916 |  | putative amino acid transporter | | -2.08 | -3.62 | 0.0071 | 0.0000 |
| PA2917 |  | probable transcriptional regulator | | -2.30 | -2.41 | 0.0024 | 0.0001 |
| PA3091 |  | hypothetical protein | | -2.43 | -1.97 | 0.0534 | 0.0547 |
| PA3104 | *xcpP* | secretion protein XcpP | | -2.44 | -2.18 | 0.0004 | 0.0001 |
| PA3278 |  | hypothetical protein | | -3.15 | -4.60 | 0.0000 | 0.0000 |
| PA3450 | *lsfA* | 1-Cys peroxiredoxin | | -2.26 | -2.61 | 0.0133 | 0.0001 |
| PA3792 | *leuA* | 2-isopropylmalate synthase | | -2.50 | -2.68 | 0.0002 | 0.0000 |
| PA4317 |  | hypothetical protein | | -2.15 | -2.33 | 0.0044 | 0.0000 |
| PA4423 |  | putative stress response protein | | -2.17 | -2.04 | 0.0036 | 0.0008 |
| **PA4525** | ***pilA*** | **major T4P pilin protein** | | **-2333.25** | **-212.52** | **0.0000** | **0.0000** |
| PA4550 | *fimU* | minor pilin protein | | -2.01 | -4.27 | 0.0480 | 0.0000 |
| PA4551 | *pilV* | minor pilin protein | | -2.01 | -4.27 | 0.0480 | 0.0000 |
| PA4552 | *pilW* | minor pilin protein | | -2.12 | -4.28 | 0.0645 | 0.0000 |
| PA4553 | *pilX* | minor pilin protein | | -2.12 | -4.28 | 0.0645 | 0.0000 |
| PA4554 | *pilY1* | putative T4P adhesin | | -1.98 | -6.30 | 0.0154 | 0.0000 |
| PA4556 | *pilE* | minor pilin protein | | -2.03 | -3.05 | 0.1052 | 0.0000 |
| PA4704 | *cbpA* | cAMP-binding protein A | | -3.36 | -2.93 | 0.0000 | 0.0000 |
| PA5027 |  | hypothetical protein | | -2.16 | -3.40 | 0.0080 | 0.0000 |
| PA5137 |  | hypothetical protein | | -2.42 | -2.89 | 0.0012 | 0.0000 |
| PA5295 |  | putative diguanylate cyclase | | -2.09 | -2.36 | 0.0057 | 0.0000 |
| PA5332 | *crc* | catabolite repression control protein | | -3.53 | -3.49 | 0.0000 | 0.0000 |
| PA5353 | *glcF* | glycolate oxidase subunit | | -2.47 | -1.82 | 0.0006 | 0.0239 |
| PA5354 | *glcE* | glycolate oxidase subunit | | -2.47 | -1.82 | 0.0006 | 0.0239 |
| PA5355 | *glcD* | glycolate oxidase subunit | | -2.71 | -2.30 | 0.0697 | 0.0600 |
| PA5472 |  | hypothetical protein | | -2.22 | -3.30 | 0.0022 | 0.0000 |
|  | |  | | --- | |  | |  |  |  |  |
| 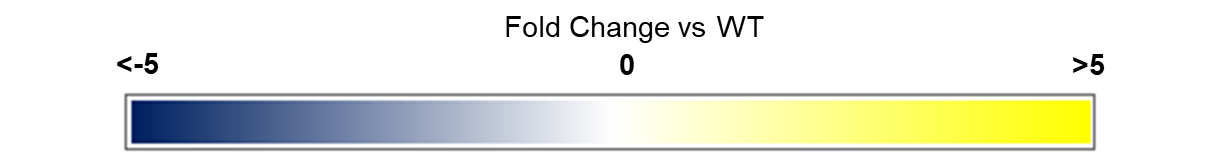 |  |  | |  |  |  |  |
|  |  |  | |  |  |  |  |
|  |  |  | |  |  |  |  |
|  | cAMP dependent genes | | |  |  |  |  |
