# Supplementary material for: The Pseudomonas aeruginosa PilSR Two-Component System Regulates Both Twitching and Swimming Motilities
Source: mBio. 2018 Jul 24;9(4):e01310-18. doi: 10.1128/mBio.01310-18 (PMC6058289; doi:10.1128/mBio.01310-18)
Supplement: TABLE S2 [file mbo004183994st2.doc]

| Table S2. Genes inversely dysregulated in *pilA* and *pilR* mutants | | | |  |  |  |
| --- | --- | --- | --- | --- | --- | --- |
|  |  |  |  |  |  |  |
| **PA Number** | **Gene Name** | **Product** | **Fold Change (*pilA/*WT)** | **Fold Change (*pilR/*WT)** | **qValue (pilA/*WT)*** | **qValue (*pilR*/WT)** |
| PA0507 |  | acyl-CoA dehydrogenase | 4.89 | -3.42 | 0.000 | 0.000 |
| PA0951a |  | unannotated | 5.17 | -64.65 | 0.039 | 0.000 |
| PA0952 |  | hypothetical protein | 4.78 | -49.02 | 0.001 | 0.000 |
| PA0952a |  | unannotated | 8.55 | -10.17 | 0.000 | 0.000 |
| PA1512 | *hcpA* | secreted protein (T6S) | 3.04 | -3.25 | 0.029 | 0.000 |
| PA4027 |  | hypothetical protein | 5.47 | -2.07 | 0.000 | 0.143 |
| PA4683 |  | hypothetical protein | 5.12 | -4.46 | 0.000 | 0.000 |
| PA5228 |  | hypothetical protein | 2.06 | -4.76 | 0.530 | 0.000 |
| PA5228a |  | unannotated | 1.92 | -9.31 | 0.772 | 0.000 |
| PA5267 | *hcpB* | secreted protein (T6S) | 2.64 | -4.96 | 0.095 | 0.000 |
|  | 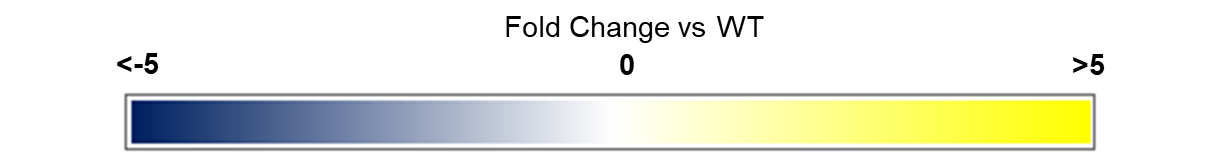 | | | | |  |
|  |  |
|  |  |
|  |  |
|  |  |  |  |  |  |  |
|  |  |  |  |  |  |  |
