# Supplementary material for: The Pseudomonas aeruginosa PilSR Two-Component System Regulates Both Twitching and Swimming Motilities
Source: mBio. 2018 Jul 24;9(4):e01310-18. doi: 10.1128/mBio.01310-18 (PMC6058289; doi:10.1128/mBio.01310-18)
Supplement: TABLE S3 [file mbo004183994st3.doc]

| Table S3. Genes dysregulated by loss of *pilR* only | | | |  |  |
| --- | --- | --- | --- | --- | --- |
|  |  |  | |  |  |
| **PA Number** | **Gene Name** | **Product** | **Fold Change (PilR/WT)** | | **q Value** |
| PA0026 | *plcB* | phospholipase C | | -2.33 | 0.000 |
| PA0050 |  | hypothetical protein | | -2.04 | 0.008 |
| PA0141 |  | conserved hypothetical protein | | -2.31 | 0.001 |
| PA0201 |  | hypothetical protein | | -2.32 | 0.000 |
| PA0295 |  | probable periplasmic polyamine binding protein | | -2.60 | 0.000 |
| PA0328 | *aaaA* | arginine-specific autotransporter | | -2.12 | 0.000 |
| PA0534 | *pauB1* | FAD-dependent oxidoreductase | | -3.16 | 0.000 |
| PA0611a |  | unannotated | | -3.21 | 0.000 |
| PA0652 | *vfr* | virulence factor regulator | | -2.07 | 0.042 |
| PA0658 |  | short-chain dehydrogenase | | -2.10 | 0.000 |
| PA0738 | *putP* | sodium/proline symporter | | -2.07 | 0.000 |
| PA0747 |  | probable aldehyde dehydrogenase | | -2.03 | 0.000 |
| PA0844 | *plcH* | hemolytic phospholipase C precursor | | -2.02 | 0.000 |
| PA0904 | *lysC* | aspartate kinase alpha and beta chain | | -2.02 | 0.001 |
| PA0912 |  | hypothetical protein | | -2.70 | 0.000 |
| PA1077 | *flgB* | flagellar basal-body rod protein | | -3.01 | 0.000 |
| PA1078 | *flgC* | flagellar basal-body rod protein | | -3.01 | 0.000 |
| PA1079 | *flgD* | flagellar basal-body rod modification protein | | -3.01 | 0.000 |
| PA1080 | *flgE* | flagellar hook protein | | -2.73 | 0.000 |
| PA1081 | *flgF* | flagellar basal-body rod protein | | -3.53 | 0.000 |
| PA1082 | *flgG* | flagellar basal-body rod protein | | -3.00 | 0.000 |
| PA1083 | *flgH* | flagellar L-ring protein precursor | | -3.00 | 0.000 |
| PA1092 | *fliC* | flagellin | | -3.91 | 0.000 |
| PA1098 | *fleS* | two-component sensor kinase (flagellar) | | -3.56 | 0.000 |
| PA1099 | *fleR* | two component response regulator | | -2.63 | 0.000 |
| PA1100 | *fliE* | flagellar hook-basal body complex protein | | -2.94 | 0.000 |
| PA1101 | *fliF* | Flagella M-ring outer membrane protein precursor | | -2.94 | 0.000 |
| PA1245 | *aprX* | Apr Type I Secretion protein | | -2.31 | 0.000 |
| PA1422 | *gbuR* | Arginine and proline metabolism protein | | -3.56 | 0.000 |
| PA1423 | *bdlA* | biofilm dispersion locus A | | -6.52 | 0.000 |
| PA1429 |  | probable cation-transporting P-type ATPase | | -3.17 | 0.000 |
| PA1441 | *fliK* | putative flagellar hook-length control protein | | -3.04 | 0.000 |
| PA1442 |  | conserved hypothetical protein | | -2.06 | 0.000 |
| PA1452 | *flhA* | flagellar biosynthesis protein | | -4.04 | 0.000 |
| PA1453 | *flhF* | flagellar biosynthesis protein | | -2.56 | 0.000 |
| PA1474 |  | hypothetical protein | | -2.00 | 0.002 |
| PA1476 | *ccmB* | heme exporter protein | | -2.01 | 0.001 |
| PA1546 | *hemN* | oxygen-independent coproporphyrinogen III oxidase | | -2.40 | 0.042 |
| PA1561 | *aer* | aerotaxis receptor | | -2.94 | 0.000 |
| PA1674 | *folE2* | GTP cyclohydrolase I | | -2.05 | 0.001 |
| PA1679 |  | hypothetical protein | | -3.60 | 0.000 |
| PA1966 |  | hypothetical protein | | -2.60 | 0.000 |
| PA1967 |  | hypothetical protein | | -3.56 | 0.000 |
| PA2027 |  | hypothetical protein | | -2.21 | 0.001 |
| PA2113 | *opdO* | pyroglutamate porin | | -2.37 | 0.001 |
| PA2114 |  | probable major facilitator superfamily transporter | | -2.37 | 0.001 |
| PA2274 |  | hypothetical protein | | 5.35 | 0.000 |
| PA2275 | *yahK* | alcohol dehydrogenase | | 2.45 | 0.039 |
| PA2399 | *pvdD* | pyoverdine synthase D | | -2.02 | 0.001 |
| PA2400 | *pvdJ* | pyoverdine synthase J | | -2.04 | 0.005 |
| PA2407 | *fpvC* | putative adhesin | | -2.09 | 0.000 |
| PA2408 | *fpvD* | putative ATPase | | -2.09 | 0.000 |
| PA2446 | *gcvH2* | glycine cleavage system protein H2 | | -2.12 | 0.001 |
| PA2490 |  | conserved hypothetical protein | | -2.75 | 0.000 |
| PA2654 |  | probable chemotaxis transducer | | -3.71 | 0.000 |
| PA2759 | *-* | hypothetical protein | | -2.29 | 0.000 |
| PA2787 | *cpg2* | glutamate carboxypeptidase | | -2.33 | 0.000 |
| PA2867 |  | probable chemotaxis transducer | | -4.07 | 0.000 |
| PA2954 |  | hypothetical protein | | -2.40 | 0.000 |
| PA2955 |  | hypothetical protein | | -3.86 | 0.000 |
| PA3336 |  | major facilitator superfamily (MFS) transporter | | -2.06 | 0.001 |
| PA3350 | *flgA* | hypothetical protein | | -2.48 | 0.000 |
| PA3417 |  | pyruvate dehydrogenase E1 component subunit alpha | | 2.80 | 0.004 |
| PA3441 |  | probable molybdopterin-binding protein | | -4.19 | 0.000 |
| PA3612 |  | hypothetical protein | | -2.76 | 0.000 |
| PA3762 |  | hypothetical protein | | -2.37 | 0.000 |
| PA3790 | *oprC* | Putative copper transport outer membrane porin | | -2.46 | 0.000 |
| PA4235 | *bfrA* | bacterioferritin | | -2.18 | 0.001 |
| PA4309 | *pctA* | chemotactic transducer | | -2.60 | 0.000 |
| PA4310 | *pctB* | chemotactic transducer | | -3.24 | 0.000 |
| PA4326 |  | hypothetical protein | | -3.18 | 0.000 |
| PA4347 |  | hypothetical protein | | -2.36 | 0.005 |
| PA4348 |  | conserved hypothetical protein | | -2.36 | 0.005 |
| PA4356 | *xenB* | xenobiotic reductase | | -2.00 | 0.005 |
| PA4520 |  | probable chemotaxis transducer | | -2.80 | 0.000 |
| PA4524 | *nadC* | nicotinate-nucleotide pyrophosphorylase | | -7.08 | 0.000 |
| PA4526 | *pilB* | T4P assembly ATPase | | -4.68 | 0.000 |
| PA4528 | *pilD* | prepilin peptidase | | -3.83 | 0.000 |
| **PA4547** | ***pilR*** | **two-component response regulator** | | **-48.82** | **0.000** |
| PA4563 | *rpsT* | 30S Ribosomal protein S20 | | -2.02 | 0.008 |
| PA4571 |  | probable cytochrome C | | -2.15 | 0.000 |
| PA4704 | *cbpA* | cAMP binding protein | | -2.24 | 0.002 |
| PA5034 | *hemE* | uroporphyrinogen decarboxylase | | -2.04 | 0.001 |
| PA5076 |  | probable binding protein component of ABC transporter | | -2.02 | 0.021 |
| PA5231 | *yhiH* | probable ATP-binding/permease fusion ABC transporter | | -2.40 | 0.001 |
| PA5232 | *yhiI* | conserved hypothetical protein | | -2.40 | 0.001 |
| PA5435 |  | probable transcarboxylase subunit | | -2.48 | 0.032 |
| PA5436 |  | probable biotin carboxylase subunit of a transcarboxylase | | -2.48 | 0.032 |
| PA5474 |  | metalloprotease | | -2.17 | 0.011 |
|  |  |  | |  |  |
|  | 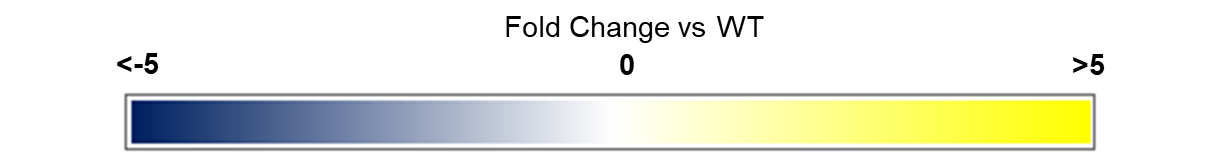 | | |  |  |
|  |  |  |
|  |  |  |
|  |  |  |
|  |  |  | |  |  |
